# Unusual Presentation of Follicular Dendritic Cell Sarcoma as a Cystic Neck Swelling

**DOI:** 10.1155/2018/4038250

**Published:** 2018-10-24

**Authors:** G. Chidananda-Murthy, P. Babu, J. Chandran, G. Raja

**Affiliations:** ^1^Sri Venkateshwaraa Medical College Hospital & Research Centre, Department of Surgical Oncology, Puducherry, India; ^2^Sri Venkateshwaraa Medical College Hospital & Research Centre, Department of Plastic Surgery, Puducherry, India; ^3^Sri Venkateshwaraa Medical College Hospital & Research Centre, Department of Pathology, Puducherry, India; ^4^Sri Venkateshwaraa Medical College Hospital & Research Centre, Department of Radiology, Puducherry, India

## Abstract

Follicular dendritic cell sarcoma is a very rare neoplasm that most commonly involves cervical lymph nodes and usually presents as a solid mass. Presentation as a cystic neck mass is very rare. Radiological studies and aspiration cytology are often unreliable, and diagnosis is usually made after thorough clinical and pathological examination along with immunohistochemical analysis. In this article, we report a case of a 42-year-old man who presented with right-sided neck swelling of a 2-month duration. Contrast-enhanced CT showed a cystic lesion in the right side of the neck inferomedial to parotid gland located between medial border of sternocleidomastoid muscle and internal jugular vein. Fine needle aspiration cytology was inconclusive. Patient underwent excision biopsy. Histological examination showed a solid-cystic tumor composed of spindle cells arranged in storiform pattern and showed a positive staining for CD23, CD35, and CD21 that confirmed the diagnosis of follicular dendritic cell sarcoma.

## 1. Introduction

Second branchial cleft cyst (SBCC) is the most common cystic swelling occurring in the neck [1]. Branchial cysts are a developmental anomaly of branchial apparatus that are present usually in the thirties. There are four different types of SBCC based on location. Type II branchial cyst is the commonest. It occurs deep to sternocleidomastoid (SCM) muscle and lies on the great vessels. It is found at the junction of upper and mid-third of SCM muscle and is usually asymptomatic but sometimes may be complicated by infection or hemorrhage [2]. Other neck lesions that present as cystic swellings include cystic lymph node metastases from head and neck squamous cell carcinomas (SCCs) and papillary thyroid carcinomas [3, 4]. Though lymph node metastases from SCC are usually solid, they can undergo cystic degeneration with a reported incidence of 30–60% [3]. Very rarely, it can be lymphoma with cystic degeneration or cystic necrotic schwannoma.

Follicular dendritic cell sarcoma (FDCS) is a very rare neoplasm arising from follicular dendritic cells [5]. It is grouped along with tumors of histiocytes and dendritic cells in the World Health Organization classification of tumors [6]. These tumors commonly occur in lymph nodes of cervical region but can also involve axillary or mediastinal lymph nodes and extranodal sites [5, 7]. They often present as solid painless neck swellings [5, 7]. To the best of the author's knowledge, presentation of FDCS as a cystic swelling has not been reported so far. In this article, the authors describe a case of FDCS presenting as a cystic neck swelling.

## 2. Case Report

**Figure 1:**
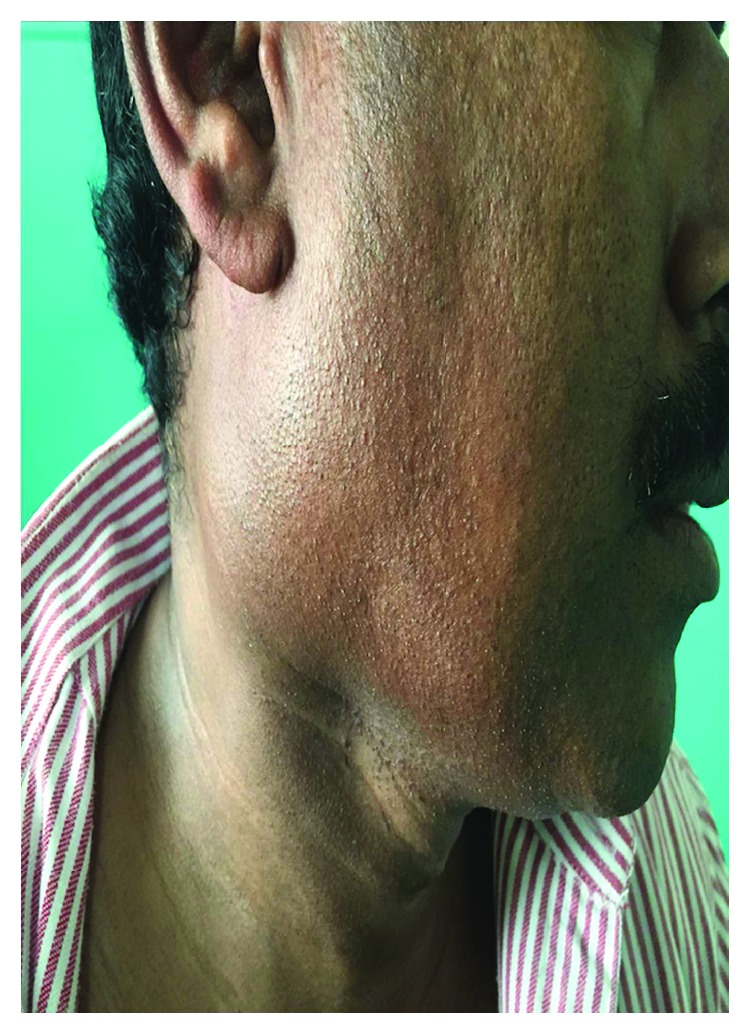
Swelling on the right side of the neck below the angle of mandible.

**Figure 2:**
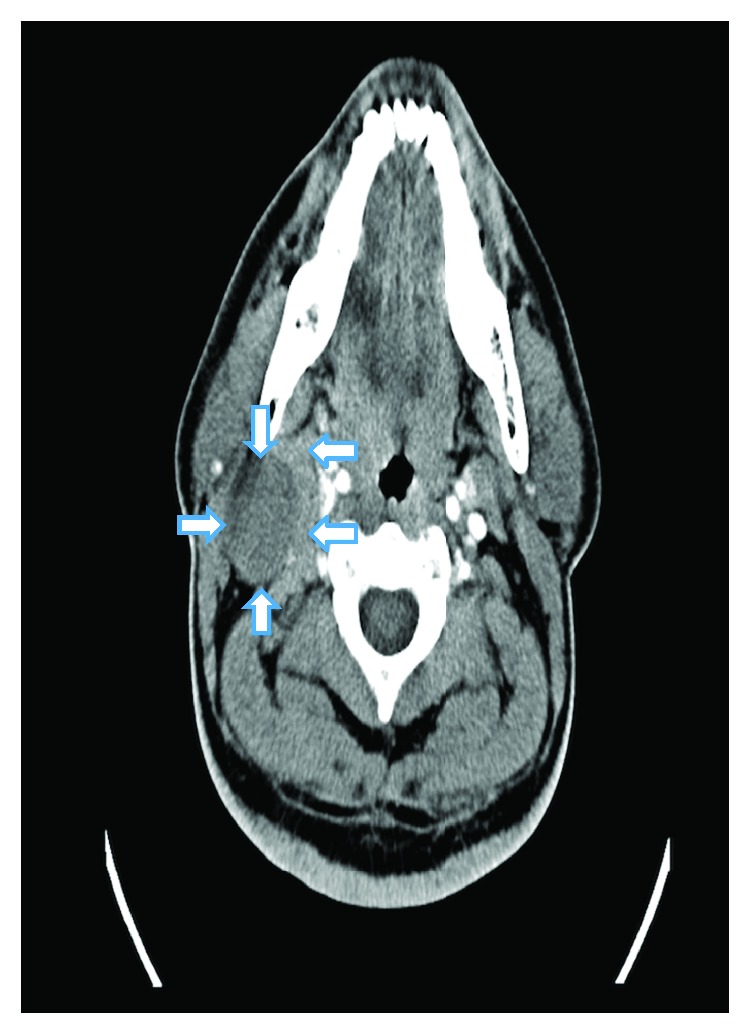
CECT neck shows cystic lesion medial to sternocleidomastoid muscle and inferomedial to parotid gland (arrows showing edge of the tumor). Eccentric solid area shows minimal enhancement on contrast administration.

**Figure 3:**
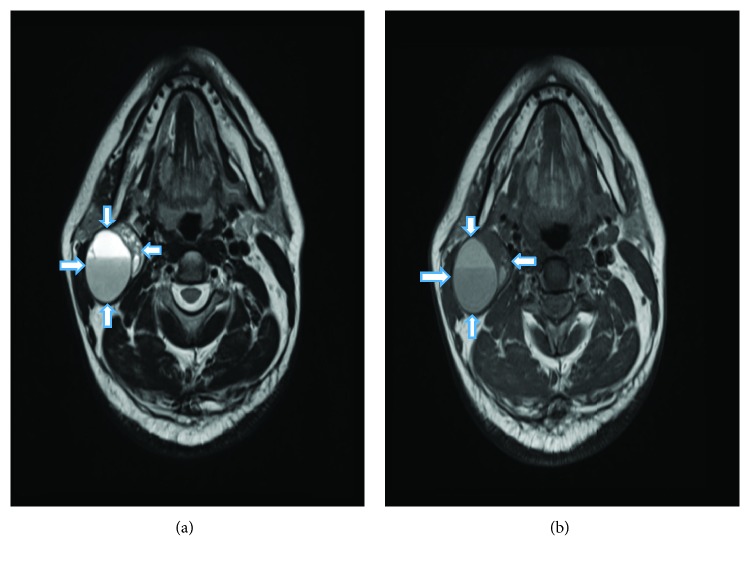
(a) T2-weighted magnetic resonance imaging shows hyperintense fluid-fluid level and isointense eccentric solid area (arrows showing edge of the tumor). (b) T1-weighted magnetic resonance imaging shows hyperintense fluid-fluid level (arrows showing edge of the tumor).

A 42-year-old man presented with swelling on the right side of the neck for two months which was insidious in onset and gradually progressive. He was asymptomatic except for a single episode of fever associated with pain in the swelling which subsided after a course of antibiotics. He was a nonsmoker and had no history of chewing tobacco, consumption of alcohol, or prior radiation exposure. On examination, there was a solitary swelling of 5 × 3 cm on the right side of the neck, below the angle of mandible which was deep to sternocleidomastoid muscle at the junction of upper and mid-third of the muscle. It was nontender, firm in consistency, and with well-defined borders and smooth surface. Skin over the swelling was normal and pinchable. It was noncompressible and nonpulsatile (Figure 1). Examination of the oral cavity and other systems was normal. Contrast-enhanced computed tomography (CECT) of the neck was performed which showed a solitary, relatively well-defined predominantly cystic lesion measuring 3.8 × 3.7 × 3.9 cm with smooth margins and minimally enhancing eccentric solid areas in the right side of the neck inferomedial to parotid gland located between sternocleidomastoid muscle laterally and carotid space medially (Figure 2). On magnetic resonance imaging (MRI), cystic component of the lesion showed fluid-fluid level that was hyperintense on both T1- and T2-weighted images suggesting hemorrhagic or proteinaceous component. The eccentric solid component was heterogenous and isointense on T2-weighted images (Figures 3(a) and 3(b)). Fine needle aspiration cytology (FNAC) of the swelling revealed hemorrhagic fluid and inconclusive cytology; hence, excision biopsy of the swelling was performed. A transverse skin incision was placed over the swelling along the upper cervical skin crease. A well-encapsulated 4 × 3 × 3 cm cystic swelling was present in the region of level II between medial border of sternocleidomastoid and internal jugular vein. Superiorly, it extended up to mastoid process. It was excised completely without any spillage. On exploration, there were no significantly enlarged lymph nodes. Postoperative period was uneventful.

**Figure 4:**
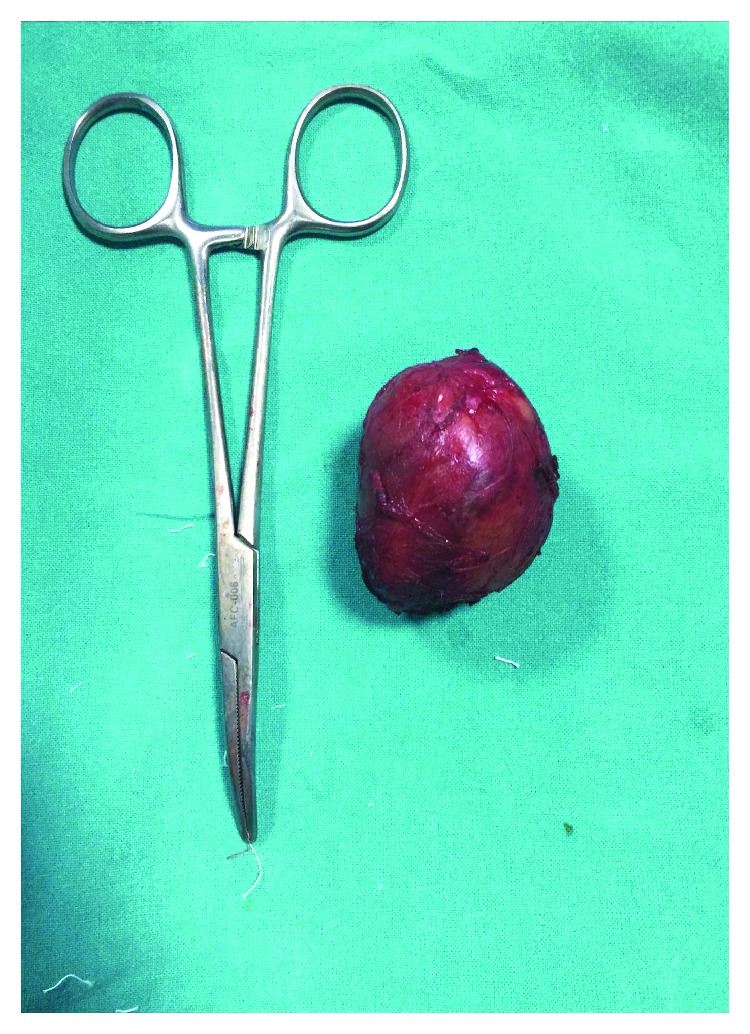
Well-circumscribed tumor with smooth surface.

**Figure 5:**
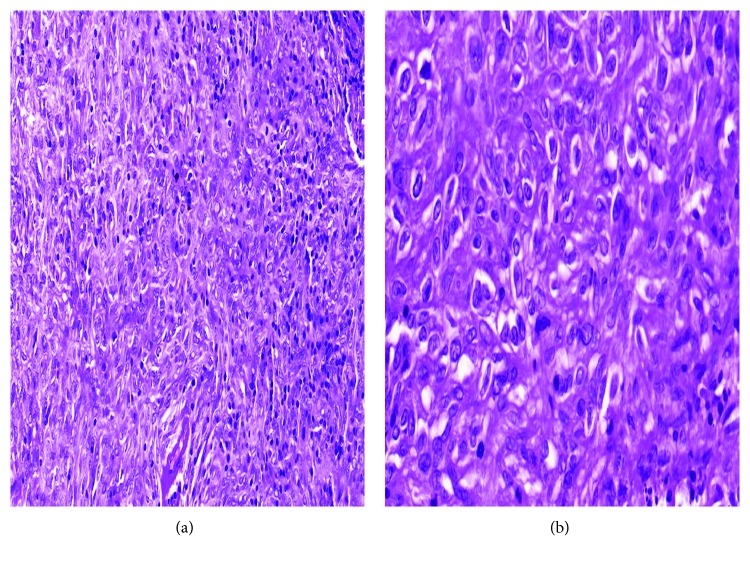
(a) Tumor cells are arranged in storiform pattern and as short fascicles. Tumor cells are intermixed with scattered lymphocytes (hematoxylin and eosin; magnification, 200x). (b) Tumor cells are oval to elongated with indistinct cytoplasmic border and ovoid nucleus with few of them showing prominent nucleoli (hematoxylin and eosin; magnification, 400x).

**Figure 6:**
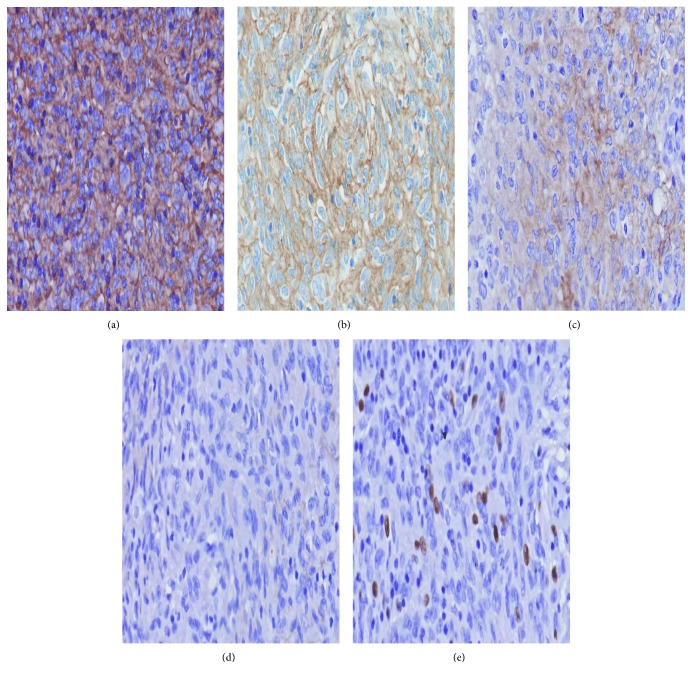
(a) Tumor cells show strong membranous staining for CD23 (magnification, 400x). (b) Tumor cells show positive staining for CD35 (magnification, 400x). (c) Tumor cells show weak membranous staining for CD21 (magnification, 400x). (d) Tumor cells show negative staining for panCK (magnification, 400x). (e) Tumor has a low mitotic activity of 4–5% (magnification, 400x).

Histological examination revealed a globular solid-cystic mass of size 4.5 × 3.5 × 2.5 cm (Figure 4). Cystic area measured 3.5 × 2.6 cm and was filled with blood while the solid area measured 2 × 1.1 × 0.8 cm. Microscopically, tumor was composed of ovoid to spindle-shaped cells arranged in sheets and focal storiform pattern (Figure 5(a)). Tumor was interspersed with numerous vascular spaces and hyalinized blood vessels. Perivascular cuffing of lymphocytes was present. Individual tumor cells had vesicular nucleus with diffuse fine chromatin (Figure 5(b)). Immunohistochemical analysis showed positive staining for CD23 (Figure 6(a)), CD35 (Figure 6(b)), and vimentin. Tumor cells showed weak staining for CD21 (Figure 6(c)) and negative staining for pan-cytokeratin (panCK) (Figure 6(d)), leukocyte common antigen (LCA), epithelial membrane antigen (EMA), and smooth muscle actin (SMA). Ki67 index was 4–5% (Figure 6(e)). Based on the histologic findings and immunohistochemistry, a diagnosis of FDCS was made. Further staging was done with positron emission tomography-CT (PETCT) that did not show any other site of involvement. The case was discussed in multidisciplinary tumor board, and no further treatment was advised since surgical resection was complete and no adverse prognostic features were present. There is no evidence of recurrence of disease after one year of follow-up.

## 3. Discussion

Follicular dendritic cell sarcoma is a very rare neoplasm arising from FDCs that was first described by Monda et al. in 1986 [8]. Follicular dendritic cells are components of B cell follicles found in the lymph nodes, spleen, and mucosa-associated lymphoid tissue. These are involved in antigen presentation, germinal center reaction generation, and regulation. Unlike dendritic cells, these are not derived from bone marrow hematopoietic stem cell but are believed to develop from a host mesenchymal precursor cell. These present antigens to T lymphocytes mainly on CD21 (CR2) and CD35 (CR1) [9].

The exact etiology of FDCS is not known. Few cases have been shown to be associated with hyalinized vascular Castleman's disease [5, 10]. It is thought that it transforms into FDCS through hyperplasia dysplasia sequence. However, clonal relationship has not been established and further research is needed [10]. A variant of FDCS has been described that resembles inflammatory pseudotumor and it is thought that Epstein-Barr virus (EBV) may have an etiological role in such tumors [5, 11].

Follicular dendritic cell sarcomas commonly occur in lymph nodes of cervical region but also in mediastinal and axillary lymph nodes [5, 7, 12]. The incidence of extranodal site FDCS is around 30–40% and includes sites such as the tonsil, palate, pharynx, mesocolon, pancreas, and small bowel with the most common being tonsils and pancreas [5, 13, 14]. Cases have been reported in the breast, lung, and retroperitoneum also [14]. These tumors occur most commonly between 35 and 45 years and do not show any sex predilection [5, 7, 12]. They usually present as painless enlarging neck swelling without any systemic manifestations [5, 7, 12]. In the present case, patient presented with painless neck mass with progressive increase in size without associated symptoms.

Very less information is available in literature regarding radiological features of FDCS. Long-Hua et al. reported that these tumors have variable and nonspecific features on CECT [15]. According to them, these tumors are usually well defined and homogenous but can have areas of low attenuation due to internal necrosis. In our case, the mass was well defined with smooth rim and no calcification or necrosis. It was predominantly cystic with a small eccentric solid component. Cystic component that was hyperintense on both T1- and T2-weighted MRI images suggested hemorrhagic or proteinaceous component. This correlated with the findings on pathological examination that showed blood-filled cystic area measuring 3.5 × 2.6 cm. Eccentric solid component showed minimal enhancement on CECT and was isointense on T2-weighted MRI with few hyperintense areas possibly due to hemorrhage. There were no other enlarged regional lymph nodes. Presentation of FDCS as a cystic swelling on CECT/MRI has not been reported till now to the best of our knowledge.

It is difficult to differentiate FDCS from commonly found cystic neck masses such as branchial cyst, cystic lymph node metastases, and neurogenic tumors with cystic degeneration based on imaging alone. Uncomplicated branchial cyst is well circumscribed with smooth rim and nonenhancing on CECT. However, wall thickening and enhancement may occur due to associated inflammation. On MRI, branchial cyst has high-signal intensity on T2-weighted images and low-signal intensity on T1-weighted images depending on protein content [1]. Cystic nodal metastases from squamous cell carcinoma are usually multiple and have focal areas of low attenuation on CECT. They have high-signal intensity on T2-weighted images and low-signal intensity on T1-weighted images on MRI [16]. Neurogenic tumors are located posterior to the neck vessels, whereas paragangliomas are located between external and internal carotid vessels causing splaying. These tumors may have cystic areas secondary to mucinous degeneration, hemorrhage, or necrosis. These tumors have smooth margins, fusiform shape, and nonhomogeneous MR signal intensities with fluid-fluid levels [16].

Diagnostic yield of FNAC of cystic swellings is very low and often this requires excision biopsy for histological confirmation [2, 3]. Grossly, these tumors are well circumscribed with pushing borders. Cut surface is tan colored with areas of hemorrhage and necrosis [5, 6]. Tumor is composed of spindle, ovoid, or polygonal cells arranged in storiform, whorled, fascicular, follicular, or trabecular pattern. Individual tumor cells contain eosinophilic cytoplasm with distinct nucleolus. The borders of tumor cells are indistinct giving a syncytial appearance. Small lymphocytes, T cells more than B cells, are interspersed throughout the tumor and are a characteristic feature of these tumors. Nuclear atypia is usually mild, and proliferation index is between 1 and 20% [5, 6]. Immunohistochemical analysis is absolutely essential for diagnosis. CD21 and CD35 are the most specific antibodies [5, 6]. However, the staining can be focal or patchy. Hence, it is preferred to use these antibodies together [5]. Reactivity for EMA, S100, desmoplakin, and vimentin is variable and not specific. These tumors are always negative for CD3, CD20, cytokeratin, and vascular markers [5, 12]. Diagnosing tumors which arise in lymph nodes is easier as lymph nodes do not contain spindle cells. But high index of suspicion is required in diagnosing those which occur in extranodal sites. Differentiating these tumors from common mesenchymal tumors is difficult and immunohistochemistry (IHC) is mandatory [13]. In our case, tumor cells were ovoid to spindle shaped arranged in storiform pattern with numerous vascular spaces and perivascular lymphocytes. On IHC, tumor cells showed positive staining for CD21, CD23, and CD35.

These tumors have a mortality rate of 15–20% [5, 7, 14]. Incidence of local recurrence is around 30–40%, and distant metastasis is around 20% [5, 7, 14]. Common sites of distant metastases are the lung, peritoneum, or liver [14]. Common prognostic factors are size of the lesion, location, and histological features such as presence of coagulative necrosis, mitotic count, and nuclear atypia. Intra-abdominal location, size > 6 cm, nuclear atypia, coagulative necrosis, and mitotic count > 5 per 10 high-power field are considered as poor prognostic factors [5, 7].

Due to the rarity of tumor, definitive recommendations regarding optimal treatment are not available. According to Chan et al. and Perez-Ordonez et al., two of the largest series, these tumors should be considered as intermediate-grade sarcoma rather than lymphoma due to their natural history and behavior [5, 7]. In most of the cases, some form of adjuvant therapy has been delivered either chemotherapy (CT) or radiotherapy (RT) or both [5, 7, 12]. Role of RT is not very clear [5]. In a pooled analysis of 97 head and neck cases by Pang et al., patients who received adjuvant RT had lower locoregional recurrence than those who did not (*p* = 0.019) [17]. Adjuvant chemotherapy is considered essential in intra-abdominal tumors and in those with poor prognostic factors [5, 7]. Chemotherapy regimen either similar to that used in high-grade lymphoma (CHOP) or sarcoma (gemcitabine and taxane or doxorubicin and ifosfamide) has been employed [5, 12, 18]. In a study by Gounder et al., there was no significant difference in prognosis in patients who received adjuvant or neoadjuvant therapy (*n* = 11) and those who underwent surgery alone (*n* = 12) [18]. Soriano et al. studied 14 patients who were treated in MD Anderson Center from 1995 to 2005. Most of the patients received adjuvant therapy, and these authors reported that those who received adjuvant CT in addition to RT had a longer disease free interval (37.6 months) than those who did not receive adjuvant CT (4.6 months) [12]. In the present case, the tumor was localized to the neck and <6 cm in size; surgical excision was complete; histologically, there was no necrosis or atypia and Ki67 index was 4–5%. Since there were no adverse prognostic factors, patient did not receive any adjuvant therapy.

## 4. Conclusion

Cystic swellings of the neck in adults are commonly presumed to be second branchial cleft cysts. This often results in inadequate evaluation of the patient and delay in surgery as well as definitive management. Radiological studies and aspiration cytology are unreliable in differentiating complicated branchial cysts from FDCS presenting as a cyst. Though such tumors are rare, they should be included in the differential diagnosis of cystic neck masses. A thorough clinical and pathological examination along with immunohistochemical analysis is essential for making a diagnosis of follicular dendritic cell sarcoma.

## References

[1] Pietarinen-Runtti P., Apajalahti S., Robinson S., Passador-Santos F., Leivo I., Mäkitie A. A. (2010). Cystic neck lesions: clinical, radiological and differential diagnostic considerations. *Acta Oto-Laryngologica*.

[2] Gold B. M. (1980). Second branchial cleft cyst and fistula. *American Journal of Roentgenology*.

[3] Flanagan P. M., Roland N. J., Jones A. S. (1994). Cervical node metastases presenting with features of branchial cysts. *The Journal of Laryngology and Otology*.

[4] Wunderbaldinger P., Harisinghani M. G., Hahn P. F. (2002). Cystic lymph node metastases in papillary thyroid carcinoma. *American Journal of Roentgenology*.

[5] Chan J. K. C., Fletcher C. D. M., Nayler S. J., Cooper K. (1997). Follicular dendritic cell sarcoma: clinicopathologic analysis of 17 cases suggesting a malignant potential higher than currently recognized. *Cancer*.

[6] Pileri S. A., Grogan T. M., Harris N. L. (2002). Tumours of histiocytes and accessory dendritic cells: an immunohistochemical approach to classification from the International Lymphoma Study Group based on 61 cases. *Histopathology*.

[7] Perez-Ordonez B., Erlandson R. A., Rosai J. (1996). Follicular dendritic cell tumor: report of 13 additional cases of a distinctive entity. *The American Journal of Surgical Pathology*.

[8] Monda L., Warnke R., Rosai J. (1986). A primary lymph node malignancy with features suggestive of dendritic reticulum cell differentiation. A report of 4 cases. *The American Journal of Pathology*.

[9] Tew J. G., Kosco M. H., Burton G. F., Szakal A. K. (1990). Follicular dendritic cells as accessory cells. *Immunological Reviews*.

[10] Chan A. C. L., Chan K. W., Chan J. K. C., Au W. Y., Ho W. K., Ng W. M. (2001). Development of follicular dendritic cell sarcoma in hyaline-vascular Castleman’s disease of the nasopharynx: tracing its evolution by sequential biopsies. *Histopathology*.

[11] Miranda R. N., Khoury J. D., Medeiros L. J. (2013). *Atlas of Lymph Node Pathology*.

[12] Soriano A. O., Thompson M. A., Admirand J. H. (2007). Follicular dendritic cell sarcoma: a report of 14 cases and a review of the literature. *American Journal of Hematology*.

[13] Hollowood K., Stamp G., Zouvani L., Fletcher C. D. M. (1995). Extranodal follicular dendritic cell sarcoma of the gastrointestinal tract: morphologic, immunohistochemical and ultrastructural analysis of two cases. *American Journal of Clinical Pathology*.

[14] Biddle D. A., Ro J. Y., Yoon G. S., Yong Y. W., Ayala A. G., Ordonez N. G. (2002). Extranodal follicular dendritic cell sarcoma of the head and neck region: three new cases, with a review of the literature. *Modern Pathology*.

[15] Long-Hua Q., Qin X., Ya-Jia G., Jian W., Xiao-Yuan F. C. (2011). Imaging findings of follicular dendritic cell sarcoma: report of four cases. *Korean Journal of Radiology*.

[16] Mittal M. K., Malik A., Sureka B., Thukral B. B. (2012). Cystic masses of neck: a pictorial review. *Indian Journal of Radiology and Imaging*.

[17] Pang J., Mydlarz W. K., Gooi Z. (2016). Follicular dendritic cell sarcoma of the head and neck: case report, literature review, and pooled analysis of 97 cases. *Head & Neck*.

[18] Gounder M., Desai V., Kuk D. (2015). Impact of surgery, radiation and systemic therapy on the outcomes of patients with dendritic cell and histiocytic sarcomas. *European Journal of Cancer*.

